# Interprofessional Collaboration on an Internal Medicine Ward: Role Perceptions and Expectations among Nurses and Residents

**DOI:** 10.1371/journal.pone.0057570

**Published:** 2013-02-28

**Authors:** Virginie Muller-Juge, Stéphane Cullati, Katherine S. Blondon, Patricia Hudelson, Fabienne Maître, Nu V. Vu, Georges L. Savoldelli, Mathieu R. Nendaz

**Affiliations:** 1 Unit of Development and Research in Medical Education (UDREM), Faculty of Medicine, University of Geneva, Geneva, Switzerland; 2 Quality of Care Service, University Hospitals of Geneva, Geneva, Switzerland; 3 Institute of Demographic and Life Course Studies, Faculty of Economic and Social Sciences, University of Geneva, Geneva, Switzerland; 4 Division of General Internal Medicine, University Hospitals of Geneva, Geneva, Switzerland; 5 Department of Community Medicine, Primary Care and Emergency Medicine, University Hospitals of Geneva, Geneva, Switzerland; 6 Division of Anaesthesiology, University Hospitals of Geneva, Geneva, Switzerland; The University of Hong Kong, Hong Kong

## Abstract

**Background:**

Effective interprofessional collaboration requires that team members share common perceptions and expectations of each other's roles.

**Objective:**

Describe and compare residents’ and nurses’ perceptions and expectations of their own and each other’s professional roles in the context of an Internal Medicine ward.

**Methods:**

A convenience sample of 14 residents and 14 nurses volunteers from the General Internal Medicine Division at the University Hospitals of Geneva, Switzerland, were interviewed to explore their perceptions and expectations of residents’ and nurses’ professional roles, for their own and the other profession. Interviews were analysed using thematic content analysis. The same respondents also filled a questionnaire asking their own intended actions and the expected actions from the other professional in response to 11 clinical scenarios.

**Results:**

Three main themes emerged from the interviews: patient management, clinical reasoning and decision-making processes, and roles in the team. Nurses and residents shared general perceptions about patient management. However, there was a lack of shared perceptions and expectations regarding nurses’ autonomy in patient management, nurses’ participation in the decision-making process, professional interdependence, and residents’ implication in teamwork. Results from the clinical scenarios showed that nurses’ intended actions differed from residents’ expectations mainly regarding autonomy in patient management. Correlation between residents’ expectations and nurses’ intended actions was 0.56 (p = 0.08), while correlation between nurses’ expectations and residents’ intended actions was 0.80 (p<0.001).

**Conclusions:**

There are discordant perceptions and unmet expectations among nurses and residents about each other’s roles, including several aspects related to the decision-making process. Interprofessional education should foster a shared vision of each other’s roles and clarify the boundaries of autonomy of each profession.

## Introduction

In an Internal Medicine ward, patient management is largely based upon interprofessional collaboration between nurses and residents. Interprofessional collaboration has been defined as “nurses and physicians working together, sharing responsibilities for solving problems, and making decisions to formulate and carry out plans for patient care” [1]. Four main components define collaboration according to the American Nurse Association [2]: a partnership with mutual valuing; the recognition of separate and combined spheres of responsibility; mutual safeguarding of legitimate interests of each party; and recognized shared goals.

In the hospital setting, interprofessional collaboration is crucial as healthcare teams face a number of challenges, such as complexity of clinical practice, high variation in clinical demand, ever-changing teams, and heavy workload. Therefore, when multidisciplinary teams experience collaboration at its best, the quality of care improves. For example, interprofessional collaboration has been associated with a lower patient death rate and reduced readmissions to the intensive care unit after patients’ transfer to the ward [1]. In a randomized, controlled study, Curley [3] showed that interdisciplinary rounds in Internal Medicine decreased patients’ length of hospital stay and costs. Other studies showed that the same positive patient outcomes are associated with enhanced nurse-physician relationships [2], [4], [5], [6].

Interprofessional perceptions of collaboration between doctors and nurses have been explored through questionnaires, interviews, focus groups or narratives written by the participants [7], [8], [9], [10], [11]. In some studies, doctors reported higher satisfaction with interprofessional collaboration than nurses [8], [12], [13]. while in other studies nurses seemed more satisfied than doctors about communication within an intensive care unit team [14]. Another study conducted in various wards of two Dutch hospitals, suggested that the quality of relational interactions was higher within professions than between professions [11]. Important differences have been shown between behaviors and role expectations among nurses and physicians [11]. For example, there is a discrepancy between the nurses’ actual behavior and their perception of their own role regarding communication with doctors and patients. There are also considerable differences between doctors’ performances with respect to psycho-social needs of patients and nurses’ expectations. According to nurses, doctors do not provide an optimal care regarding patients’ psycho-social needs. This may have negative consequences for interprofessional collaboration between both professional and thus affect the quality of care [11]. In the collaborative process, mutual respect and a match between the respective roles and expectations of doctors and nurses are required for communication, cooperation and contribution to a common goal [15]. To our knowledge, studies have more explored multidisciplinary teams in anesthesiology, emergency, and intensive care settings [16], [17], [18], [19] than in Internal Medicine.

The aim of this study was to describe and compare residents’ and nurses’ perceptions and expectations of each other’s professional roles in the setting of a hospital Internal Medicine ward, in order to identify aspects to be emphasized in future interprofessionnal education programs.

## Methods

The design of this study was a partially mixed, sequential approach, according to Leech typology [20]. It included a main qualitative approach based on thematic content analysis [21] and a quantitative aspect.

This research was approved by the research ethics committee of the University Hospitals of Geneva, which waived our protocol to enter a complete ethical review. Participants received a written description of the project and gave a written consent for participation and for the use of audio-recorded material. The absence of use of any data for summative purposes and the anonymity of the participants was guaranteed.

### Setting and Participants

The study was conducted in the Division of General Internal Medicine at the University Hospitals of Geneva, Switzerland, a 2000-bed public institution. This division encompasses 130 beds across 8 acute-care wards and one intermediate-care ward. A population of 33 residents and 54 nurses were eligible at the time of the study. The proportion of females was respectively 55% and 70%. The project was part of a multi-step study, which included an analysis of clinical reasoning during high-fidelity simulation. It was presented in regularly scheduled staff meetings involving nurses and residents for recruitment. Volunteer participants were included if they met the following inclusion criteria: residents with one to five years of experience in the Internal Medicine residency program and staff nurses actively working on the Internal Medicine ward. Using the data saturation approach, recruitment continued throughout the data collection until no new themes/concepts were emerging. Overall, we recruited 14 pairs of nurses and residents.

### Data Collection

All data were collected during a single confidential encounter with each participant. Interviews took place in a quiet room of the hospital at the end of the participants’ regular shifts, with the approval of the hospital hierarchy. Participants were asked to first fill in a brief questionnaire regarding their socio-demographic characteristics (age, gender, year of diploma, country of education, rate of employment, years of experience, years of experience in the Division of Internal Medicine). Next, the interviewer (VMJ) conducted a semi-structured interview with the participant. Finally, participants were asked to respond to a questionnaire with 11 clinical scenarios.

#### a. Semi-structured interviews

Semi-structured interviews were conducted by one researcher (VMJ) to explore participants’ perceptions and expectations of residents’ and nurses’ professional roles in Internal Medicine. The interview guide (Box S1) was pretested with two nurses and two physicians not taking part in the study.

#### b. Short clinical scenarios

Participants were given a questionnaire of 11 short clinical scenarios (examples in Box S2). For each scenario, they had to choose among 6 proposed actions either their own intended actions or the expected actions from the other professional. For each scenario, participants could choose more than one action but were asked to indicate which one they would choose in priority.

For the first six scenarios, nurses were asked to indicate their intended actions and residents were asked to indicate the actions they expected from nurses. Proposed actions were: 1) call the emergency team, 2) call the resident in charge of the patient, 3) call the chief resident, 4) call the head nurse, 5) wait for next scheduled medical round, and 6) deal with the situation oneself.

For the remaining five scenarios, residents were asked to indicate their intended actions and nurses were asked to indicate the actions they expected from residents. Proposed actions were: 1) call another resident on the ward, 2) call the chief resident, 3) call the emergency team, 4) contact the patient’s family, 5) call a medical specialist, and 6) deal with the situation oneself.

### Analysis

#### a. Semi-structured interviews

The semi-structured interviews were audio-recorded, transcribed verbatim and analyzed qualitatively using thematic content analysis.

All seven authors independently read the first five interviews, identifying key themes and issues, with a focus on identifying the roles and responsibilities that respondents associated with each of the professions. We then compared and discussed our observations of these interviews, and developed a consensus catalog of initial codes. Additional transcripts were then coded by one author (VMJ) using Atlas.ti (ATLAS.ti Scientific Software Development GmbH, Version 6.2.18), and cross-checked by two authors (SC and MN).

#### b. Short clinical scenarios

For each of the 11 scenarios, we computed the number of times each proposed action was respectively chosen by residents and nurses. We computed Spearman’s rho coefficients to assess the correlation between the choices of nurses and residents. We also analyzed the residents’ and nurses’ priority choices and assessed the proportion of them who chose to manage the case themselves or who decided to refer immediately to someone else (exact chi-square tests).

## Results

### Sample Description

The 14 residents were mostly men (male to female ratio 10∶4), whereas the 14 nurses were predominantly women (male to female ratio 4∶10) (Table 1). Mean age of the participants was 34 years (residents 31 years, nurses 37 years). On average, residents had less postgraduate experience (4 years versus 10 years for nurses) although the mean number of years in the Division of General Internal Medicine was similar (residents 3 years, nurses 4 years). Most nurses (10) had received their nursing degree outside Switzerland, in France for the vast majority.

**Table 1:** Participant characteristics.

	Residents (n = 14)	Nurses (n = 14)	Total (n = 28)
Mean age (range)	31 (25; 36)	37 (27; 48)	34 (25; 48)
Gender (N females:N males)	4∶10	10∶4	14∶14
Mean year of diploma (range)	2006 (2001; 2010)	2000 (1984; 2008)	2003 (1984; 2010)
Country of education*	CH (10); Other (4)	CH (4); Other (10)	CH (14); Other (14)
Mean rate of employment (%) (range)	100	90 (80; 100)	90 (80; 100)
Mean years of experience (range)	4 (0.5; 7)	10 (2; 25)	7 (0.5; 25)
Mean years of experience in the Divisionof Internal Medicine (range)	3 (0.5; 5)	4 (0.5; 13)	3 (0.5; 13)

* CH = Switzerland.

The 28 interviews lasted on average 25 minutes (SD = 8, range 12–48).

### Semi-structured Interviews: Role Perceptions and Expectations

Three main general themes referring to both professions (patient management, clinical reasoning and decision-making processes, and roles in the team) emerged from the interviews and were used as a framework to describe and compare nurses’ and residents’ perceptions about their roles (Tables S1 and S2).

During the interviews, participants also spontaneously reported expectations from the other profession that were not adequately met (Table 2).

**Table 2:** Unmet expectations.

	Nurses’ expectations unmet by residents	Residents’ expectations unmet by nurses
**Patient management**	–	–
		Shared decision making
		Establish a common goal for patient management
**Clinical reasoning and decision-making processes**		Understand the clinical situation
		Recognize, anticipate problem
		Verify prescriptions and medical decisions
		Exchange information
	Explain to nurses	
	Work in team	
**Teamwork**	Listen to nurses	
	Consider nurses’ opinion	
	Recognize nurses’ work	
	Availability	
	To know more about each other’s profession	

#### a. Roles in patient management

Nurses and residents shared a common overall perception of each other’s roles regarding patient management.

Residents’ roles for patient management were to perform a global and multidisciplinary approach to patient management, to inform patients about their condition and to provide explanations about their diagnoses and management. Nurses and residents agreed that the main role of a resident was to prescribe medical orders, to treat and to take care of patients.

R2: The proper role of the doctor is to have a global vision about the patient.

N5: The doctor prescribes things, he thinks more about what should be done, which drug to give, when, and why to do a given test.

Nurses’ and residents’ perceptions of nurses’ roles regarding patient management were to ensure the follow-up of the patients throughout the hospital stay, to provide psychological support to patients as well as specific nursing cares. Nurses’ role was also committed to execute medical orders, to treat and to take care of patients.

R13: Nurses see patient three or four times a day: “Oh, Mrs So-and-so, why are you sad, what’s up, it’ll all turn out fine”. So they will have this companion-like approach, or moral support, that we don’t have.

N8: Our role as nurses starts with some observation, which can lead to a discussion. I don’t know if he wants to talk about certain things that affect him at that time. And then, we apply what we know how to do, it’s a result of our own observations and assessments.

Nurses stressed the importance of their “own” professional role including nursing care according to Virginia Henderson’s principles [22], [23]: patient follow-up and development of proximity with patients. They accentuated the distinction between their “own” role and their “delegate” role, in which they execute medical orders. Residents also underlined the same specific roles of nurses’ for the quality and safety of patient care.

N1: My role, well in two points: the first one is my autonomy, which is proper to nursing care. Then comes patient care through medical delegation.

R6: The most important is for us each to have our set of skills, yet we are equal in each of our fields. I don’t know how to put in an IV. There are many things that I don’t know how to do, that the nursing staff does a thousand times better than me.

#### b. Roles in clinical reasoning and decision-making processes

Both professions agreed that one major role of residents consisted of performing reasoning and decision making, which required extensive medical knowledge and its application to the patient’s problems. Many stressed the contrast between residents’ scientific knowledge leading to a decision-making process and nurses’ competence to bring the decision into action through their know-how.

N12: They are at the core of the decisions to change treatment plans, so we won’t point them towards a given treatment because that’s something they’re trained to do. But we provide the results, we bring them the findings that might make them say “we could change this treatment”, which is then integrated with blood tests, and the clinical exam. So they still are the central person for decisions.

R6: Sometimes we are more the head and the nursing staff is more the hands but it does not mean there is a hierarchical gradient.

N6: Maybe I’ll dare to draw a sort of parallel with an architect and a worker on the construction site? The architect can build a house, except that he usually isn’t the one pouring in the cement, stacking up the cinder blocks and things like that. And the worker is not necessarily there to do calculations for weights, beams, or other things for structural works. For the practical aspects of building, however, he’s the one who stirs the cement.

Residents reported a series of unmet expectation regarding nurses' active participation in the decision-making process for patient management (Table 2). They expected nurses to have a better understanding of the clinical situation and to share the process of decision making. In their opinion, nurses should be more skilled at recognizing and anticipating the patient problems during daily management. Finally, nurses should verify the medical prescriptions and decisions and make suggestions more actively and more often. The residents also mentioned how complex it could be to establish common goals with nurses for patient management.

R2: When I sometimes express to nurses that two options are possible, their irritating answer is: “Well, I don’t know, I am not the doctor”. Well, no participation in the decision-making process, it gets tiresome.

R1: Patient management is mainly our duty but we also rely on nurses to report some problems. They also have a say about the patient’s management and suggestions to make.

Nurses also indicated that it is important for themselves to understand the clinical situation and to verify residents’ prescriptions and medical decisions.

N5: We are here to execute doctors’ orders, and also to think about whether to find anything inadequate or inappropriate.

Overall, nurses seemed satisfied with their role regarding decision-making process while residents expected more involvement from nurses.

#### c. Roles in the team

Nurses and residents shared a common overall representation of their roles in the team and emphasized the importance of working as a team, communicating, and exchanging information.

R9: Well, I think we work hand in hand in the end, so we have the same goals, we have the same job, give or take a few details.

N13: The doctor’s role is to collaborate with the whole team. The doctor who sees the patient without considering the opinions of other providers, without thinking about logistics, or feasibility for the rest of the care-team. What if we did our part without taking into account the doctor? That wouldn’t make sense. There is an important collaboration there, of course.

Both professions stressed the necessity of residents to take nurses’ opinions into consideration.

R11: It’s true that if we make the effort to explain things to nurses when they don’t understand something or when we make the effort to listen to them and to share our reflection with them, I believe that it is not only positive for patient care but it is also completely good perceived by nurses, the fact that we take into account their opinion, because we also need it finally.

N14: The doctor’s role is also to take our opinion into consideration for patient decisions, his future, or general attitude for resuscitation, end of life, etc.

Residents and nurses also shared the concept of complementing each other, but nurses positioned themselves as a link between the patient and the doctor.

R3: It’s our role to be on one side of the bridge, and the patient on the other, but without the bridge, it’s hard to communicate.

R13: We complement each other, but we don’t have the same function. So responsibilities aren’t the same: there’s the decision-making, then the application of the decision and there are other completely different things that are separate. For example, helping patients bathe, that’s their job entirely, and I never take part in that.

However, nurses felt that the residents did not work enough in team, that their opinions about treatment choices or application were not enough taken into consideration, and that residents did not listen to them sufficiently (Table 2). They expected more recognition of their work from residents. Additionally, nurses expected to be given more medical explanations regarding the patient’s illness or the treatment they had to administer. As a consequence, nurses wished more availability of the residents, although all recognized that residents have a high workload and little spare time.

N3: Very often, we are with residents who are close to and who listen to us. But it is not always the case. Sometimes we are treated like scenery, they don’t ask our opinion.

N13: So the doctor’s role is to prescribe, and to take care of the patient, but in his own way, let’s say, all the while communicating well with the patient, being as close to the patient as they can. They have trouble doing this because they’re a little overwhelmed, these brave doctors.

Both professions estimated that nurses depended on residents for actions that are not related to strict nursing care. However, residents felt that they are heavily dependent on nurses and cannot work without them, especially regarding the patient follow-up and concrete patient care. Yet nurses perceived that residents could be independent and able to perform nurses’ clinical actions if they had enough time.

R3: Doctors and nurses are like husband and wife, one cannot do something without the other if one wants to build something good.

N2: The doctor gives orders and I will apply them, this is the big difference. I cannot accomplish my work without the doctor. The doctor can manage without the nurse, but he would probably miss some time to get some information.

Finally, all residents and nurses aspired to know more about each other’s profession.

N8: I don’t think I know all the details about doctor’s role, but I do see it in what he does. But I’ve never asked a doctor to explain his role to me. And since I’ve never been through medical school, I just don’t know.

R8: I think that doctors, we don’t always know what a nurse needs to do during a day of work, which is why we don’t necessarily interact with her enough to address her expectations.

### Short Clinical Scenarios

In the short clinical scenarios, the subjects had to indicate their own intended actions and the actions they expected from the other profession.

We summarized the number of times each proposed action was chosen across the 11 written scenarios, respectively by residents and nurses (Table S3). The overall correlation of action choices between nurses and residents was 0.68 (p<0.001). For scenarios concerning residents’ actions, the correlation between nurses’ expectations from residents and residents’ intended actions was 0.80 (p<0.001). For scenarios concerning nurses’ actions, correlation between residents’ expectations from nurses and nurses’ intended actions was 0.56 (p = 0.008). Nurses’ intended actions differed from residents’ expectations mainly regarding nurses’ autonomy in patient management, reflected by the low correlation (0.36) regarding the action “deal with the situation oneself”. Residents expected to be paged by the nurses while nurses estimated to be able to start the management of the situation by themselves. Such differences were not found in cases concerning residents’ actions.

Analyses performed only on the priority action intended or expected (Table 3) confirmed that residents expected nurses to call for help in priority while nurses often estimated to be able to start patient management by themselves.

**Table 3:** Priority action choices across 11 short clinical scenarios.

6 short clinical scenarios: actions intended by nurses and expected by residents*		
	Residents	Nurses
Call somebody	54% (45/84)	38% (32/84)
Manage him/herself	46% (39/84)	62% (52/84)
p = 0.04		
**5 short clinical scenarios: actions intended by residents and expected by nurses** *		
	**Residents**	**Nurses**
Call somebody	54% (37/69)	62% (39/63)
Manage him/herself	46% (32/69)	38% (24/63)
p = 0.34		

* The denominators (84, 69, 63) correspond to the number of valid responses through the cases mentioned as priority actions by the participants.

These findings contrasted with the results of the interviews during which the residents expected more autonomy from nurses in patient management.

## Discussion

### Interpretation of Results in Relation to Other Studies

Effective interprofessional collaboration requires common shared perceptions and expectations of each team member’s role [11], [15], [19]: Collaboration between residents and nurses contributes to the quality of teamwork, a necessary condition for optimal patient care [2], [3], [4], [5], [6], [24]. Overall, the perceptions of residents and nurses in our study were similar to many items of the Jefferson Scale of Attitude toward Physician–Nurse Collaboration which are deemed important by a group of nursing students [25]. These items include nurses’ responsibility in patient care and follow-up, suggestions to the physicians, psychological support to the patient, collaboration with physicians rather than mere assistance, and common education to understand their respective roles. Overall, the domains mentioned by our participants meet the four components of the nurse-physician collaboration published by the American Nurse Association [2]: partnership with mutual valuing, recognition of separate and combined spheres of responsibility, mutual safeguarding of legitimate interests of each party and recognition of shared goals.

There was a shared perception of the traditional roles of physicians and nurses regarding patient management. According to the subjects, physicians possess the medical knowledge to solve the patient’s problem and they are the ones who prescribe tests and treatments that are then carried out by nurses [1]. They also communicate with patients and families, and are responsible for the global and coordinated patient management. Besides their delegated roles, nurses stressed their own specific role, often described according to the Virginia Henderson dimensions [22], [23]. They take care of the basic needs of the patients and provide them support. They are often considered as the links between patients and physicians and are expected to ensure regular patient follow-up and monitoring. Nurses spontaneously emphasized these roles during the interviews, suggesting their need to clarify the boundaries of their professional identity and of their autonomy [26].

The perceptions and expectations among nurses and residents were discordant for several aspects. The first one is about clinical reasoning and decision-making processes. Interestingly, the perceptions during the interviews differed from the actions chosen in the clinical scenarios. During the interviews, residents expected more involvement from nurses in the medical decision process, anticipation, and proaction. Yet they did not give nurses credit for their autonomy in patient management during the clinical scenarios, since they expected to be paged by the nurses. Conversely, nurses expressed their autonomy in the initial management of the patient in the clinical scenarios. Baggs and Weller [1], [19] found similarly that residents often endorsed the entire responsibility for decision making. Reeves [27] pointed out that interactions between physicians and other caregivers consisted mostly of unidirectional requests for information or tasks, with minimal opportunities for discussion.

Contrarily, in the interviews, residents expected nurses to be involved in the decision-making process in many ways: sharing decision making, establishing common goals for patient management, understanding the clinical situation, recognizing and anticipating patient’s problems, and making suggestions. This reflects Snelgorve’s study [28] which found that residents generally valued nurses who had more clinical experience than themselves. However, at the time of intended actions in the clinical scenarios, they seemed to be less able to apply these views in practice and automatically reverse to the position in which physicians take the responsibility of the decision-making process. This discrepancy may explain the negative perception of nurses on various aspects of decision making in the teamwork with physicians [13]. Alternatively, it could be understood as a consequence of the status of residents, who on one hand have medical authority on nurses, but on the other hand often have less experience than nurses.

In Weller’s study [19], nurses wanted some involvement in decision-making process beyond executing orders. They felt that they could be more useful if residents listened to their suggestions. In our interviews, the majority of nurses only mentioned that they should understand the clinical situation and verify prescriptions and medical decisions although they did not explicitly mention their need to be involved in the decision-making process. If nurses feel that residents are not taking their suggestions into account, as it arose from our data about teamwork, they will probably not perceive their role as decision makers. Thus, it seems that nurses intend to have more autonomy in patient management than expected by residents but have difficulty applying it in practice. This could be due either because they do not feel sufficiently listened by residents or perhaps because they feel the residents' contradictory attitudes towards nurses' autonomy, as observed in our data. However, increasing opportunities for nurses to participate in the decision-making process seems important since it could lead to better patient outcomes [29].

Another aspect with unmatched perceptions and expectations refers to aspects of teamwork. Nurses expected residents to provide more explanations about patient treatment and patient problems, more involvement in teamwork, more listening and consideration of their opinions and more recognition of their work. While residents spontaneously perceived most of these roles there seems to be a hiatus between residents’ intentions and nurses’ expectations as described in several studies about team perceptions of interprofessional collaboration [8], [12], [13], [14]. For example, in Thomas’s cross-sectional surveys [13], while only 33% of the nurses rated collaboration and communication with physicians as high or very high, 73% of physicians rated collaboration and communication with nurses as high or very high. These findings reflect our data because nurses are less satisfied with interprofessional teamwork due to several expectations unmet by residents. As recommended in the Report of the Interprofessional Education Collaborative and by the World Health Organization [30], [31], actively listening and encouraging ideas and opinions from other team members are essential components of patient care. To promote collaboration, it is important to allow discussion and consider others’ opinions [1]. If nurses feel that their inputs are not heard by residents, they will probably make fewer suggestions, while residents expect more participation. Thus, there is a risk to enter a vicious circle in which expectations are less and less met, leading to poor teamwork. These findings may reflect issues related to hierarchy, site of training and culture, all known to affect teamwork attitudes [13], [14], [32].

Both professions reported their limited knowledge about the others’ profession and their wish to learn more about it. This may account for some unmet expectations, because the relevance of what can be expected from someone is closely related to the missions of each profession. The need for improved knowledge about each other’s roles and responsibilities has been reported in the literature [1], [19], [30]. Effective collaboration can occur only when professionals are aware of their complementarities. A limited understanding of roles and responsibilities of team members could have an impact on appropriate task distribution. Discussions between nurses and residents could be a way to clarify each member’s roles and responsibilities in order to modify their actual behavior in practice [11], [30].

### Strengths and Weaknesses of the Study

One strength of our study was our mixed-method design, which allowed us to improve our vision on nurses’ autonomy, since the discrepancy between interviews and clinical scenarios would not have been detected by one method alone. Our study had the particularity of involving residents and nurses working in Internal Medicine, a context which has not been widely studied until now. Interviews were conducted by a non-healthcare professional (an educator) to avoid hierarchical relationships between investigator and participants. Additionally, the interpretation of the data was also performed by investigators with medical background.

This study took place in only one hospital setting and may not be representative of other hospitals or departments, although we felt we reached saturation regarding the main themes raised during the interviews after we interviewed 14 pairs of subjects. However, this low number of subjects may limit the strength of the statistical analyses of the quantitative approach of this study. Additionally, participants volunteered for the study and were thus potentially interested in the topic, which may have biased the content of their speech. Finally, the male:female ratio in our sample was higher than the ratio in our eligible resident population, while both ratios were similar for nurses. This may limit the representativeness of residents’ perceptions.

### Implications

Interprofessional education is recommended at both undergraduate and postgraduate levels [14], [33], [34], [35], leading many institutions to set up various projects. According to our findings, several dimensions need to be addressed in such educational projects for the sake of relevance: interprofessional education should foster a shared vision and understanding of each other’s roles and clarify the boundaries of autonomy of each profession. It should also take into account the underlying culture, for example regarding hierarchical rapports, which may also influence the successful implementation of such training.

### Future Research

The differences between nurses’ and residents’ perceptions and expectations suggest a number of areas for future research. How do the perceptions and expectations of their hierarchy fit or influence their own perceptions? How do their role perceptions influence their reasoning approach to the patient’s problem? Do participants’ perceptions of their own role fit their actual performance in simulated situations? Some of these questions will be addressed in the next stages of our research.

## Supporting Information

Table S1 Perceptions of nurses’ roles
(DOCX)

Table S2 Perceptions of residents’ roles
(DOCX)

Table S3 Number of times each proposed action was chosen across 11 short clinical scenarios
(DOCX)

Box S1 Interview guide used for nurses and residents
(DOCX)

Box S2 Examples of short clinical scenarios
(DOCX)
