# Supplementary material for: Interprofessional Collaboration on an Internal Medicine Ward: Role Perceptions and Expectations among Nurses and Residents
Source: PLoS One. 2013 Feb 28;8(2):e57570. doi: 10.1371/journal.pone.0057570 (PMC3585159; doi:10.1371/journal.pone.0057570)
Supplement: Table S1 — Perceptions of nurses’ roles (DOCX) [file pone.0057570.s001.docx]

**Table S3: Perceptions of nurses’ roles**

|  | **By nurses mainly** | **By nurses and residents** | **By residents mainly** |
| --- | --- | --- | --- |
|  |  | Follow-up, patient psychological support, proximity to patients |  |
| **Patient management** |  | Delegate role (execute orders) |  |
|  |  | Treat, care |  |
|  |  | Nursing care |  |
|  |  |  | Shared decision making |
|  |  |  | Establish a common goal for patient management |
| **Clinical reasoning and decision-making processes** |  |  | Recognize, anticipate problem |
|  |  | Understand the clinical situation |  |
|  |  | Verify prescriptions and medical decisions |  |
|  |  |  | Make suggestions |
| **Teamwork** |  | Work in team, communicate, exchange information, inform |  |
|  | Are dependent on residents |  |  |
