# Supplementary material for: Interprofessional Collaboration on an Internal Medicine Ward: Role Perceptions and Expectations among Nurses and Residents
Source: PLoS One. 2013 Feb 28;8(2):e57570. doi: 10.1371/journal.pone.0057570 (PMC3585159; doi:10.1371/journal.pone.0057570)
Supplement: Table S2 — Perceptions of residents’ roles (DOCX) [file pone.0057570.s002.docx]

**Table S4: Perceptions of residents’ roles**

|  | **By nurses mainly** | **By nurses and residents** | **By residents mainly** |
| --- | --- | --- | --- |
|  |  | Global patient management and coordination (multidisciplinary) |  |
| **Patient management** | Inform, explain to patients |  |  |
|  |  | Prescribe orders |  |
|  |  | Treat, care |  |
| **Clinical reasoning and decision-making processes** |  | Apply medical knowledge, perform decision-making process |  |
|  |  | Explain to nurses |  |
|  |  | Work in team, communicate, exchange information, inform |  |
| **Teamwork** |  | Are overloaded and unavailable |  |
|  | Listen to nurses |  |  |
|  |  | Consider nurses’ opinion |  |
|  |  | Validate, recognize nurses’ work |  |
|  |  |  | Are dependent on nurses |
|  |  |  |  |
