# Supplementary material for: Interprofessional Collaboration on an Internal Medicine Ward: Role Perceptions and Expectations among Nurses and Residents
Source: PLoS One. 2013 Feb 28;8(2):e57570. doi: 10.1371/journal.pone.0057570 (PMC3585159; doi:10.1371/journal.pone.0057570)
Supplement: Table S3 — Number of times each proposed action was chosen across 11 short clinical scenarios (DOCX) [file pone.0057570.s003.docx]

**Table S5: Number of times each proposed action was chosen across 11 short clinical scenarios**

| **6 short clinical scenarios: actions intended by nurses and actions expected by residents** | | | |
| --- | --- | --- | --- |
| **Actions** | **Nurses intended actions (mean/case, SD)** | **Residents expected actions from nurses (mean/case, SD)** | **Correlation*** |
| 1. Nurse calls the emergency team | 1 (0.07, 0.26) | 0 | - |
| 2. Nurse calls the resident in charge of the patient | 46 (3.29, 1.49) | 55 (3.93, 1.26) | 0.74 |
| 3. Nurse calls the chief resident of the unit | 1 (0.07, 0.26) | 1 (0.07, 0.26) | - |
| 4. Nurse calls the unit head nurse | 13 (0.93, 0.26) | 18 (1.29, 0.73) | 0.99 |
| 5. Nurse waits for the scheduled next medical round | 20 (1.43, 1.22) | 27 (1.93, 1.14) | 0.50 |
| 6. Nurse deals with the situation oneself | 53 (3.79, 1.42) | 30 (2.14, 2.21) | 0.36 |
| **Overall** |  |  | **0.56 (p=0.008)** |
| **5 short clinical scenarios: actions intended by residents and actions expected by nurses** | | | |
| **Actions** | **Nurses expected actions from residents (mean/case, SD)** | **Residents intended actions (mean/case, SD)** | **Correlation*** |
| 1. Resident calls another resident on the ward | 4 (0.29, 0.82) | 1 (0.07, 0.27) | - |
| 2. Resident calls the chief resident | 38 (2.71, 1.27) | 30 (2.14, 1.41) | 0.82 |
| 3. Resident calls the emergency team | 12 (0.85, 0.66) | 16 (1.14, 0.77) | 0.99 |
| 4. Resident contacts the patient’s family | 1 (0.07, 0.26) | 5 (0.35, 0.74) | 0.50 |
| 5. Resident calls a medical specialist | 12 (0.85, 1.02) | 12 (0.85, 0.77) | 0.95 |
| 6. Resident deals with the situation oneself | 20 (1.42, 1.15) | 28 (2, 0.78) | 0.67 |
| **Overall** |  |  | **0.80 (p<0,001)** |

*Spearman’s rho across cases
