# Supplementary material for: Interprofessional Collaboration on an Internal Medicine Ward: Role Perceptions and Expectations among Nurses and Residents
Source: PLoS One. 2013 Feb 28;8(2):e57570. doi: 10.1371/journal.pone.0057570 (PMC3585159; doi:10.1371/journal.pone.0057570)
Supplement: Box S1 — Interview guide used for nurses and residents (DOCX) [file pone.0057570.s004.docx]

**Box S1: Interview guide used for nurses and residents**

1. What is your role (nurse or resident) in the ward?

2. What is the role of the other professional (nurse or resident) in the ward?

3. In your opinion, what are the differences between nurses’ and residents’ roles? And what are the similarities?

4. What are the typical moments of interaction with the other professional (nurse or resident) during a working day?

5. Could you describe one positive situation of collaboration that you experienced? What were the factors facilitating interprofessional collaboration?

6. Could you describe one situation of more difficult collaboration that you experienced? What were the factors preventing interprofessional collaboration?
