# Supplementary material for: Interprofessional Collaboration on an Internal Medicine Ward: Role Perceptions and Expectations among Nurses and Residents
Source: PLoS One. 2013 Feb 28;8(2):e57570. doi: 10.1371/journal.pone.0057570 (PMC3585159; doi:10.1371/journal.pone.0057570)
Supplement: Box S2 — Examples of short clinical scenarios (DOCX) [file pone.0057570.s005.docx]

**Box S2: Examples of short clinical scenarios**

**Example of a clinical scenario asking nurses for their intended actions:**

A 65 year-old male patient, admitted for a myocardial infarction, has been doing well for the past 4 days. He rings the call button and complains of chest pain; he is sweaty and uncomfortable. Blood pressure 140/70 mmHg, irregular pulse at 120/min, respiratory rate of 24/min.

How do you deal with this situation? You:

- - Call the emergency team
  - Call the resident in charge of the patient
  - Call the chief resident of the unit
  - Call the unit head nurse
  - Wait for the next scheduled medical round
  - Deal with the situation yourself: Describe your action(s).

**Example of a clinical scenario asking residents for their expected actions from nurses:**

A 65 year-old male patient, admitted for a myocardial infarction, has been doing well for the past 4 days. He rings the call button and complains of chest pain; he is sweaty and uncomfortable. Blood pressure 140/70 mmHg, irregular pulse at 120/min, respiratory rate of 24/min.

How should the nurse deal with this situation? She/he should:

- - Call the emergency team
  - Call the resident in charge of the patient
  - Call the chief resident of the unit
  - Call the unit head nurse
  - Wait for the next scheduled medical round
  - Deal with the situation him/herself: Describe your action(s).

**Example of a clinical scenario asking residents for their intended actions:**

A 61 year old male patient with a history of chronic obstructive pulmonary disease (COPD), has just been admitted for bronchopneumonia. He is treated with corticosteroids, nebulizations, and antibiotics. This morning the patient rang the call button because he was having trouble catching his breath just sitting on the edge of his bed. After lunch, he is found lying in his bed but is difficult to wake up. His respiration rate is 30/min. The nurse calls you.

What do you do at this point? You:

- - Call another resident on the ward
  - Call the chief resident
  - Call the emergency team
  - Contact the patient’s family
  - Call a medical specialist
  - Deal with the situation yourself

**Example of a clinical scenario asking nurses for their expected actions from residents:**

A 61 year-old male patient with a history of chronic obstructive pulmonary disease (COPD), has just been admitted for bronchopneumonia. He is treated with corticosteroids, nebulizations, and antibiotics. This morning the patient rang the call button because he was having trouble catching his breath just sitting on the edge of his bed. After lunch, he is found lying in his bed but is difficult to wake up. His respiration rate is 30/min. You call the unit’s resident physician.

What do you think the resident should do at this point?

- - Call another resident on the ward
  - Call the chief resident
  - Call the emergency team
  - Contact the patient’s family
  - Call a medical specialist
  - Deal with the situation him/herself
